# Molecular Profiling of the Lateral Habenula in a Rat Model of Depression

**DOI:** 10.1371/journal.pone.0080666

**Published:** 2013-12-05

**Authors:** Trine Christensen, Line Jensen, Elena V. Bouzinova, Ove Wiborg

**Affiliations:** Translational Neuropsychiatry Unit, Department of Clinical Medicine, Aarhus University, Risskov, Denmark; INSERM/CNRS, France

## Abstract

**Objective:**

This study systematically investigated the effect of chronic mild stress and response to antidepressant treatment in the lateral habenula at the whole genome level.

**Methods:**

Rat whole genome expression chips (Affymetrix) were used to detect gene expression regulations in the lateral habenula of rats subjected to chronic mild stress (mild stressors exchanged twice a day for 8 weeks). Some rats received antidepressant treatment during fifth to eights week of CMS. The lateral habenula gene expression profile was studied through the gene ontology and signal pathway analyses using bioinformatics. Real-time quantitative polymerase chain reaction (RT-PCR) was used to verify the microarray results and determine the expression of the *Fcrla, Eif3k, Sec3l1, Ubr5, Abca8a, Ankrd49, Cyp2j10, Frs3, Syn2, and Znf503* genes in the lateral habenula tissue.

**Results:**

In particular we found that stress and antidepressant treatment affected intracellular cascades like growth factor receptor signaling, G-protein-coupled receptor signaling, and Wnt signaling – processes involved in the neuroplastic changes observed during the progression of depression and antidepressant treatment.

**Conclusion:**

The present study suggests an important role of the lateral habenula in the development of depression-like conditions and correlates to previous studies demonstrating a significant role of the lateral habenula in depressive-like conditions and antidepressant treatment.

## Introduction

Depression is a major cause of disability affecting about 350 million people worldwide. However, little is known about the underlying pathology of the disease. The complexity of the disease is massive involving several neurological mechanisms and affecting numerous regions of the brain. The therapeutic intervention of depression-associated symptoms has evolved rapidly over the past two decades. Nevertheless, currently available antidepressants have significant limitations, including slow onset of action and low rates of response including treatment refraction. Despite substantial advances in understanding depression symptomatology, as well as the antidepressant machinery, the underlying molecular mechanisms still remain unclear [1].

The lateral habenula (LHb) is a small brain structure mediating behavioral responses to pain, stress, anxiety, sleep and reward and its dysfunction is associated with depression, schizophrenia and drug-induced psychosis [2], [3]. Accumulating lines of evidences indicate that the LHb, which innervates multiple brain regions and directly influences the serotonergic, noradrenergic and dopaminergic brain systems, exhibits hyperactivity during depressed states. Increased neuronal activity of the habenula has been observed in animal models of stress [4] and depression [5], [6], and also in human depression [7], [8]. Conversely, lesions of the LHb were shown to induce antidepressant-like effects in rats exposed to inescapable foot shock (learned helplessness) [9] and chronic mild stress (CMS) [10]. Similarly, neuronal inhibition of the LHb by deep brain stimulation has shown ameliorating effects in the CMS model of depression [11] while pharmacological inhibition of the LHb mediates an antidepressant effect in an animal model of treatment resistant depression [12]. Indeed, one study reports antidepressant effects of deep brain stimulation in a treatment resistant depressed patient [13].

The CMS model of depression was developed to mirror anhedonia – one of the core symptoms of depression. Anhedonia is characterized as a lack of interest in otherwise pleasurable events or stimuli, and in the CMS model of depression this stress-induced decrease in reward sensitivity is measured as a reduction in sucrose consumption/preference [14]. The model fulfills all standard validity criteria and has a face validity that includes several symptoms of depression [15]. The CMS model of depression has been validated in several studies linking anhedonic-like behavior to deficits in cognitive behavior [16], neuroendocrine and immunological dysfunctions [17], [18], alterations at a cellular and structural level [19], [20], and dysfunctions in serotoninergic and GABAergic neurotransmitter systems [21], [22]. Additionally, several studies indicate specific gene- and protein expression profiles associating with anhedonic-like behavior [23]–[27]. The validity of the CMS model of depression is further strengthen by the fact that the model in addition to anhedonia also mirrors the aspect of stress-resilience, as a fraction (about 20%) of the stress exposed rats does not reduce their sucrose intake. Furthermore, antidepressant treatment mediates reversing effects of the stress-induced decrease in sensitivity to reward in about 50% of the treated rats, resulting in a drug responder group and drug non-responder group, respectively [23]–[25], [27]. Hence the CMS model of depression not only simulates the chronic and episodic aspect of clinical depression, but it also models the fact that conventional antidepressant therapy is inefficient in a substantial fraction of treated patients [28], [29].

In the present study we investigated global transcriptomic profiles of CMS phenotypes to search for biomarkers/molecular mechanisms underlying vulnerability to depression (anhedonia) and the long-term action of antidepressant treatment. The aim was to clarify the role of the LHb in depression and antidepressant treatment at a molecular level. The results of the present study indicate that stress-induced anhedonia and antidepressant treatment primarily associated with changes in intracellular signaling mechanisms in the LHb.

## Materials and Methods

### Ethics statement

All procedures involving animals were accepted by the Danish National Committee Ethics in Animal Experimentation (2008/561–477) and approved by the Aarhus University review board.

### Animals

Male Wistar rats were purchased from Taconic, Denmark. The initial experimental group contained 329 rats. 249 rats were submitted to CMS while the rest (80 rats) were used as unchallenged control rats. After four weeks, rats exposed to CMS were divided according to their hedonic status evaluated by decrease in sucrose intake. Thirty six rats were used in the present large-scale transcriptomic study, nine of which were stress-unchallenged controls treated with vehicle. Eighteen from 27 anhedonic-like CMS rats were exposed to escitalopram (5 mg/kg/day) treatment. Nine rats were injected with saline and used as treatment controls. 

The animals were singly housed, except when grouping was applied as a stress parameter. Food and water was available *ad libitum* except when food and/or water deprivation was applied as a stress parameter. The standard 12-h light/dark cycle was only changed in course of stress regime.

During the CMS protocol all animals with symptoms of anhedonia demonstrated decreased weight gain and cognitive impairments as was shown repeatedly before [16], [18], [19].

### Sucrose consumption test

The animals were first habituated to consume a palatable sucrose solution (1.5%). The habituation period lasted for five weeks. In this period, the sucrose test was made twice a week during the first three weeks and once a week during the last two weeks. Animals were food and water deprived 14 h prior to the test, which was a one hour period with free access to a bottle of the sucrose solution. During the stress period the sucrose consumption test was performed once a week.

### Chronic mild stress protocol

On the basis of sucrose consumption in the two final baseline tests, the animals were divided into two matched groups and placed in separate rooms. One group was exposed to an initial four weeks of chronic mild stressors and the other one was left undisturbed. The stress procedure was performed according to a procedure optimized in our laboratory [19]. Briefly, rats were submitted to one period of intermittent illumination, stroboscopic light, grouping, food or water deprivation; two periods of soiled cage and no stress; and three periods of 45° cage tilting. During grouping, rats were housed in pairs with different partners, with the individual rat alternately being a resident or an intruder. All the stressors lasted from 10 to 14 hours.

Following exposure to stress, rats were characterized as being anhedonic-like (defined as a >30% within-subject decrease in sucrose intake, also denoted as an anhedonic index value of 0.70 or less when compared to baseline) or resilient (defined as a <10% within-subject decrease in sucrose intake). Rats not responding to either criterion were excluded from the experiment. Stress-resilient rats were not included in the present study. The proportion of anhedonic-like, stress-resilient and intermediate grouped rats is shown in a recent publication by Henningsen et al. [26].

After the initial four weeks of exposure to stress, the stress group was divided into two matched subgroups and subjected to chronic escitalopram and vehicle administration, respectively, for four weeks. Stress was continued during the entire period of treatment. During treatment, the drug-treated groups segregated into drug responders and drug non-responders. We have in a previous study shown that escitalopram-treated CMS animals follow a bimodal segregation into two subgroups of responders and non-responders based on readouts from all four weeks of treatment [19].

Drug or vehicle was administered intraperitoneally once a day in the morning. Escitalopram (Sequoia Research Products Limited, United Kingdom) was dissolved in saline and given at dosages of 5 mg/kg/day.

Following the exposure of antidepressant treatment rats were characterized as drug responders (defined as a≥10% within-subject increase in sucrose intake, if the readout is above the anhedonic index value of 0.70) or drug non-responders (defined as <10% increase in sucrose intake). Rats not responding to either criterion were excluded from the experiment. Individual CMS groups (n = 9) were divided into three subgroups (n = 3) (Table 1).

**Table 1:** Animal groups investigated in the present CMS study.

Animal groups in CMS	Abbreviation	No. of animals in CMS groups	No. of subgroups for microarray
Unchallenged control vehicle	U-V	9	3 (n = 3 in each subgroup)
CMS vehicle	CMS-V	9	3 (n = 3 in each subgroup)
CMS escitalopram responders	Esc-R	9	3 (n = 3 in each subgroup)
CMS escitalopram non-responders	Esc-NR	9	3 (n = 3 in each subgroup)

n – number of rats in each group resulting in 3 subgroups per CMS group.

### Tissue processing

Rats were decapitated and brains were removed and immediately frozen on dry ice. Frozen brains were sectioned coronally (i.e. −2.12 to −4.16 mm relative to Bregma; [30] on a cryostat (CM3050S, Leica Microsystems, GmbH, Germany). 50 µm sections were collected throughout the lateral habenula. Immediately after sectioning, each tissue section was mounted on polyethylene napthalate (PEN) glass slides (Molecular Devices, USA) and stored at −80°C until further processing.

### Tissue staining

Slides were removed from −80°C storage and placed in 96% ethanol for one minute. Tissue sections were then stained in 1% cresyl violet (CV) (dissolved in absolute ethanol) for 15–25 s. Slides were then dehydrated in graded alcohols; 30s in 96% ethanol, 30 s in absolute ethanol and finally one minute in a second ethanol (absolute) step. Tissue sections were air-dried for a couple of minutes prior to laser capture microdissection (LCM).

### Laser capture microdissection

The LCM technique was used to isolate homogeneous LHb tissues devoid of contamination from the surrounding thalamic and medial habenular tissues. LCM was performed using the Veritas Microdissection Instrument model 704 (Molecular Devices, USA) with CapSure Macro caps (Molecular Devices, USA). The LHb was visualized in the microscope of the LCM instrument and captured by the “cut & capture” feature. The location of dissected area is demonstrated in Figure S1. The LCM procedure was recently described in a previous study [31]. Optimized settings were 80 mW pulse power, 3.500 µsec pulse duration, 45 µm laser spot diameter, and a UV laser power at 15. The LHb area was selected for capture using the 2× objective while the capture-part was performed on the 20× objective. Captured LHb tissue was removed from the caps by use of a pipette and placed in a 0.5 ml PCR tube containing 20 µl QIAzol buffer (Qiagen Inc., USA). The sample was centrifuged at 14.500 rpm for 30 s and stored at −80°C until RNA extraction.

### RNA isolation and quality control

After tissue homogenization, total RNA was isolated from each subgroup by use of miRNeasy Mini Kit (Qiagen Inc., USA) according to the manufacturer's protocol. Prior to the microarray analyses, the isolated total RNA was assessed to confirm high quality with respect to integrity and purity. The RIN (RNA integrity number) value of our RNA samples, as measured by the 2100 BioAnalyzer (Agilent Technologies, USA) was 7 or higher, indicating high integrity of the total RNA used for downstream analyses. The quantity of RNA samples was measured by use of Nanodrop ND-1000 (Thermo Scientific, USA).

### RNA amplification and microarray hybridization

In this study, gene expression profiles of rats exposed to CMS and antidepressant treatment (four different groups – table 1) were determined using whole genome-wide gene expression microarray analysis. All groups contained three biological replicates, therefore, a total of 12 microarrays were analyzed. Tissue from three individual rats was included in each biological replicate. Samples (15ng total RNA) were reverse transcribed and amplified using the Ribo-SPIA^®^ Ovation™ Pico WTA system V2 (NuGEN, USA) according to the manufacturer's instructions. 5 μg cDNA was used for fragmentation and labeling by use of the FL-ovation™ cDNA biotin module (NuGEN, USA) according to the manufacturer's instructions. Labeled cDNA samples of 4 μg were hybridized to the Affymetrix GeneChip^®^ High-throughput (HT) RG-230 Perfect Match (PM) 24-Array Plate for rat (Affymetrix, USA). Each array probes approximately 28.700 genes and contains more than 31.000 probes. Affymetrix Expression Console Software (Affymetrix, USA) was used for normalization (Probe Logarithmic Intensity Error Estimation – PLIER normalization strategy) of microarray data. Microarray results are available in the ArrayExpress database: accession E-MTAB-1857.

### Verification of regulated genes in the lateral habenula by real-time qPCR

TaqMan® qPCR analysis was performed by AROS, ApS (Aarhus, Denmark) using Fluidigm® technology. All selected RNA specimens were the same as the ones used in the chip experiment. Primer sets and probes for 13 tested genes were obtained from Life Technologies (filial Denmark, Naerum, Denmark). NCBI gene classification and context sequences for the tested genes are shown in table S1. The standard curves for each gene amplification were used for quantification of gene expression. The average slope of curves was −3.31±0.07 demonstrating a high efficiency of all performed qPCR reactions.

### Cluster Analysis

Cluster analysis was used to separate the four different groups based on between-groups similarities in global gene expression profiles. The clustering approach orders objects in a treelike structure and provides information about relations among groups. The cluster analyses were performed with Cluster software version 2.11 [32]. The first cluster analysis was based on all probes being expressed by the cDNA microarray chip. The second cluster analysis was based on the most regulated genes (13.976 genes) based on all four CMS groups calculated as standard deviation/mean (individual gene transcript intensity) > standard deviation/mean (all gene transcript intensities). Data were adjusted by log_2_ transformation, normalization and mean center of genes and arrays. The hierarchical clustering was performed by clustering of arrays followed by average linkage clustering. Subsequently, the cluster analysis was visualized by Tree View software version 1.60 (http://rana.lbl.gov/EisenSoftware.htm).

### Statistical analyses

**Figure 1:**
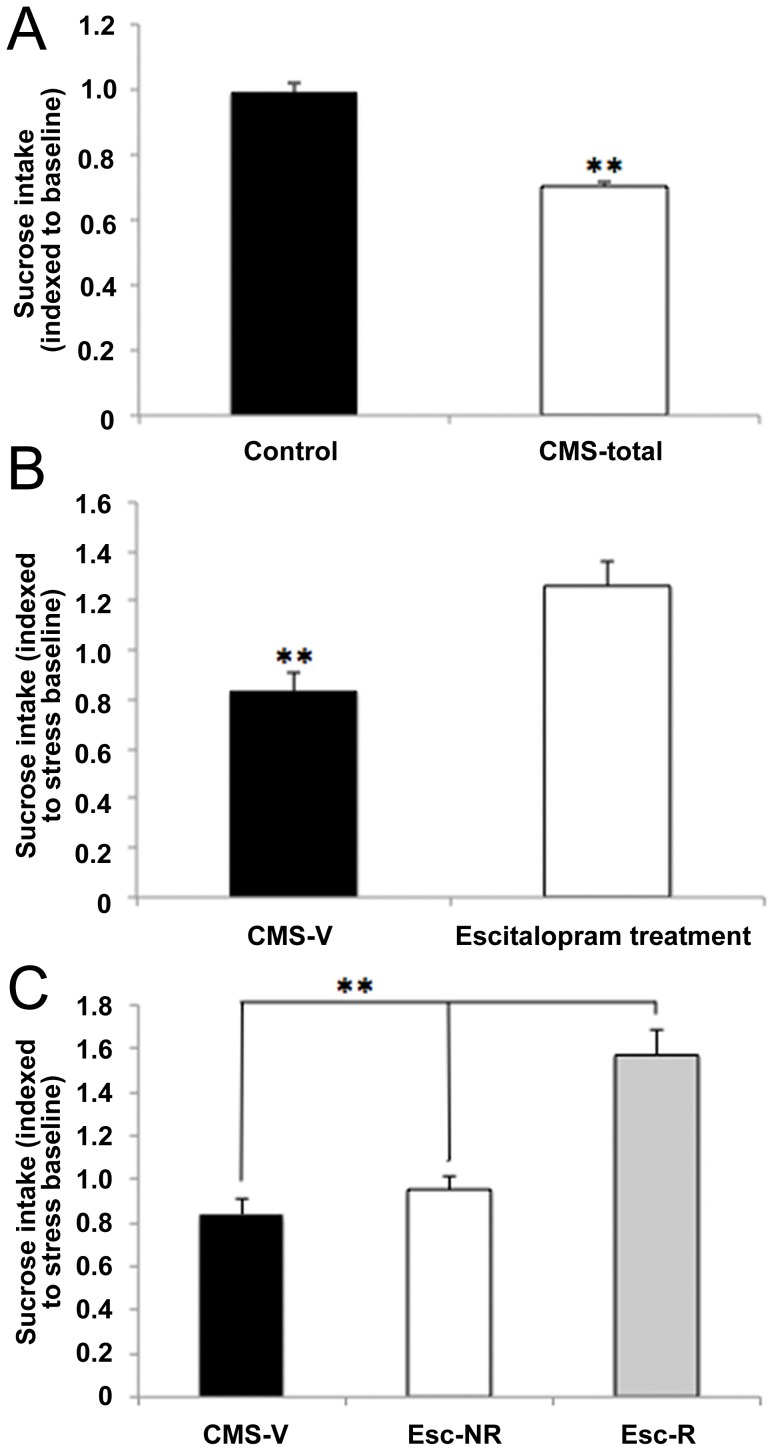
Effects of stress and treatment, respectively. (A) Four weeks exposure to chronic mild stress (CMS rats, *n* = 249) resulted in a significantly decreased sucrose consumption when compared to unchallenged controls (*n* = 80). (B) Four weeks exposure to antidepressant treatment with escitalopram resulted in a significantly increased sucrose consumption when comparing all escitalopram-treated CMS rats (responders and non-responders, respectively, *n* = 18) with vehicle-treated anhedonic-like CMS-rats (CMS-V, *n* = 9). (C) Four weeks of treatment with escitalopram resulted in a significant increase in sucrose consumption in a subgroup of drug-treated rats, i.e. rats responding to escitalopram (Esc-R, *n* = 9) treatment. A subgroup of the treated rats did not increase in sucrose intake (CMS escitalopram non-responders, Esc-NR, *n* = 9). Statistical differences were set at *p<0.05 and **p<0.001. Data are shown as mean sucrose intake, indexed to baseline (before stress) and stress baseline (before treatment), respectively + standard error of mean.

Overall stress effects and treatment effects, respectively, were investigated using Student's t-tests. Stress effects were estimated by comparing all stressed rats in the trial to all non-stressed rats (Figure 1A). Treatment effects were estimated by comparing all drug-treated rats (both responders and non-responders) to non-treated rats (Figure 1B). Data obtained from weekly sucrose tests were analyzed using one-way multivariate analysis of variance (MANOVA). Antidepressant effects were investigated separately, with stress and treatment, respectively, as between subject factors and time (weeks) as within subject factor. According to the statistically significant interactions revealed by MANOVA, additional analyses were performed by one-way analysis of variance (ANOVA) to reveal time-specific differences caused by stress- or treatment. Bonferroni corrections were performed in order to account for multiple ANOVAs being run. Significant ANOVAs were followed up with groupwise Bonferroni post-hoc tests. The statistical level of significance was set at *p*<0.05.

Differential changes in gene expression were investigated by Student's t-test to reveal significant differences. However, due to a high content of low regulations (with maximum regulations of 30–40%), significant genes (*p*<5%) were investigated collectively according to biological pathways.

Changes in gene expression detected by qPCR where analyzed in XLSTAT by one-way ANOVA using two-sided Dunnett's post-hoc comparison to identify significant differences between groups.

### Functional pathway analysis of differentially regulated genes

Genes that were significantly altered by CMS and antidepressant treatment were functionally classified into cellular pathways using the Protein ANalysis THrough Evolutionary Relationships (PANTHER, www.pantherdb.org) system. This system classifies genes on the basis of their functions taking published experimental evidences and otherwise evolutionary relationships to predict functions. To assess the statistical enrichment or over-representation of these cellular pathways from our dataset relative to the global set of rat genes, a binomial statistics within the PANTHER system was applied. The statistical level of significance in PANTHER was set at *p*<0.05. Genes investigated for functional pathway analyses had to fulfill a certain criterion; the gene had to be significantly (p<5%) regulated between relevant groups (Table 2). Pools of significant genes were thus submitted to functional pathway analysis in PANTHER in order to look for pathways implicated in stress and antidepressant responses. Several pathways were shown to be implicated in stress and antidepressant responses. In order to limit the amount of pathways in the present study, pathways were only of interest if they were implicated in both the induction and recovery of depression. As illustrated in table 3, pathways have to be significantly changed when comparing U-V to CMS-V, but also when comparing CMS-V to Esc-R. The two distinct pathway regulation patterns in table 3, indicates whether pathways are affected when comparing the unchallenged healthy state (U-V) to a recovered state (Esc-R). Examples of genes within the individual pathways, affected by stress and treatment (Esc-R), respectively, are listed in table 4.

**Table 2:** Functional pathway analysis by the Protein Analysis through Evolutionary Relationships (PANTHER) system.

Groups investigated for functional pathway analyses	No. of probe sets submitted to PANTHER pathway analysis	No. of mapped probe sets by PANTHER
U-V vs. CMS-V	766	343
CMS-V vs Esc-R	722	298
Esc-R vs. Esc-NR	1375	562

Number of probe sets submitted to PANTHER (www.pantherdb.org) reflect genes being significantly regulated (p<5%) between the relevant groups. Number of mapped probe sets indicates probe sets being mapped by PANTHER and used for functional pathway analysis.

**Table 3:** Biological pathways implicated in depression/recovery (Figure 5A–B).

Recovery	Pathway Category	Pathway
Figure 5A	Growth factor receptor signaling	VEGF signaling
		EGF receptor signaling
		Angiogenesis
	G protein (G_q_) – coupled receptor signaling	Histamine H1 receptor signaling
		Oxytocin receptor mediated signaling
	G protein – coupled receptor signaling	Wnt signaling
		Glutamine glutamate conversion
		Transcription regulation by bZIP transcription factor
		P53 pathway by glucose deprivation
Figure 5B		Integrin signaling
		Parkinson disease

Classification of pathways using the Protein ANalysis THrough Evolutionary Relationships (PANTHER, www.pantherdb.org) system. Statistical enrichment of biological pathways was assessed by binomial statistics within the PANTHER system.

**Table 4:** Pathway categories implicated in recovery.

Recovery-related Pathways	No. of genes (U-V vs. CMS-V)	No. of genes (CMS-V vs. Esc-R)	Examples of genes (gene symbol; gene name)
Growth factor receptor signaling	10	10	***Stat3, 4, 6;*** Signal transducer and activator of transcription 3, 4, 6; ***Vegfa;*** Vascular endothelial growth factor A; ***Flt1;*** Vascular endothelial growth factor receptor 1; ***Pdgfrb;*** Beta-type platelet-derived growth factor receptor; ***Prkca, Prkd3, Prkci;*** Protein kinase C (alpha-type, D3, iota-type ;***Sos1;*** Son of sevenless homolog 1
P53 pathway by glucose deprivation	5	2	***Prkab2, Prkag1, Prkag2;*** 5′-AMP-activated protein kinase (subunit beta-2, subunit gamma-1, subunit gamma-2)*; * ***Ppp2cb;*** Serine/threonine-protein phosphatase 2A catalytic subunit beta
Wnt signaling (G protein-coupled receptor signaling	9	12	***Axin1;*** Axin1; ***Ppp2cb;*** Serine/threonine-protein phosphatase 2A catalytic subunit beta; ***Ppp3r1;*** Calcineurin subunit B type 1; ***Sfrp2;*** Secreted frizzled-related protein 2; ***Dvl1;*** Segment polarity protein disheveled homolog DVL-1
G protein (G_q_) – coupled receptor signaling	3	3	***Prkca, Prkd3, Prkci;*** Protein kinase C (alpha-type, D3, iota-type); **Plcd4;** 1-phosphatidylinositol-4,5-bisphosphate phosphodiesterase delta-4; ***Gnb1*** **;** Guanine nucleotide-binding protein G(I)/G(S)/G(T) subunit beta-1; ***Gng10;*** Guanine nucleotide-binding protein G(I)/G(S)/G(O) subunit gamma-10
Glutamine glutamate conversion	1	1	***Glud1;*** Glutamate dehydrogenase 1, mitochondrial
Transcription regulation by bZIP transcription factor	3	3	***Creb3l2;*** Processed cAMP-responsive element-binding protein 3-like protein 2; ***Gtf2f1, Gtf2a2;*** General transcription factor (IIF subunit 1, IIa subunit 2)
Integrin signaling	8	6	***Itga7, Itgam;*** Integrins (alpha-7 light, alpha-M); ***Fyn;*** Proto-oncogene tyrosine-protein kinase Fyn; ***Cav1;*** Caveolin-1; ***Fn1,*** Fibronectin; ***Sos1;*** Son of sevenless homolog 1
Parkinson disease	6	10	***Tor2a;*** Torsin-2A; ***Sept1, Sept2;*** Septin-1A, −2A; ***Hspa2a,*** ** ***Hspa1b, Hspa8, Hspa1l;*** Heat shock 70 kDa proteins (2a, 1b, 8, 1l); ***Psma1, Psma4, Psmb3;*** Proteasomes (alpfa type-1, alpha type-4, 1L)

U-V, unchallenged control rats; CMS-V, CMS vehicle; Esc-R, CMS escitalopram responders; Esc-NR, CMS escitalopram non-responders.

Similarly, numerous pathways are implicated in treatment responses. The amount of pathways affected by antidepressant treatment (CMS-V versus Esc-R) is reduced by selecting pathways with concomitant changes in escitalopram responding rats (Esc-R) versus escitalopram non-responding rats (Esc-NR), thus to focus on pathways related to treatment response, but with diverging effects in drug-responders (EscR) and drug-non-responders (Esc-NR), respectively. The two regulation patterns in table 5 indicate whether pathways are affected when comparing the anhedonic-like state (CMS-V) with rats not responding to antidepressant treatment with escitalopram (Esc-NR). Examples of genes within the individual pathways related to treatment response (Esc-R and Esc-NR respectively) are listed in table 6.

**Table 5:** Biological pathways implicated in treatment response (Figure 5C–D).

Treatment response	Pathway Category	Pathway
Figure 5C	Growth factor receptor signaling	VEGF signaling
	G protein – coupled receptor signaling	Metabotropic glutamate receptor II
		Metabotropic glutamate receptor III
		Endothelin signaling
		Enkephalin release
		GABA-B receptor signaling
		5HT1 type receptor mediated signaling
		Dopamine receptor mediated signaling
		Opioid prodynorphin
		Opioid proenkephalin
		Opioid proopiomelanocortin
	-	Hedgehog signaling
	-	P53 pathway by glucose deprivation
	-	Integrin signaling
	-	Ubiquitine proteasome
	-	Carnitine metabolism
	-	Carnitine and CoA metabolism
Figure 5D	Growth factor receptor signaling	FGF signaling
		EGF receptor signaling
		Angiogenesis
	G protein – coupled receptor signaling	Heterotrimeric G-protein signaling pathway-Gi alpha and Gs alpha mediated pathway
	-	Alzheimer disease – amyloid secretase
	-	Inflammation mediated by chemokine and cytokine signaling
	-	Parkinson disease

Statistical enrichment of biological pathways was assessed by binomial statistics within the PANTHER system.

**Table 6:** Pathway categories implicated in treatment response.

Treatment response-related pathways	No. of genes (CMS-V vs. Esc-R)	No. of genes (Esc-R vs. Esc-NR)	Examples of genes (gene symbol; gene name)
Growth factor receptor signaling	11	27	***Vegfa;*** Vascular endothelial growth factor A; ***Sos1;*** Son of sevenless homolog 1; ***Frs3;*** Fibroblast growth factor receptor substrate 3; ***Pik3ca;*** Phosphatidylinositol 3-kinase catalytic subunit alpha; ***Pik3r1;*** Phosphatidylinositol 3-kinase regulatory subunit alpha; ***Mapk4;*** Mitogen-activated protein kinases 4; ***Stat3;*** Signal transducer and activator of transcription 3
G protein-coupled receptor signaling	10	21	***Prkar1a;*** cAMP-dependent protein kinase type I-alpha regulatory subunit; ***Adcy2, 3, 5;*** Adenylate cyclase type 2, 3, 5; ***Gnai3;*** Guanine nucleotide-binding protein G(k) subunit alpha*;* ***Gnb1*** **;** Guanine nucleotide-binding protein G(I)/G(S)/G(T) subunit beta-1; ***Gng10;*** Guanine nucleotide-binding protein G(I)/G(S)/G(O) subunit gamma-10; ***Creb1*** **;** cAMP response element-binding protein
P53 pathway by glucose deprivation	2	10	***Ppp2cb;*** Serine/threonine-protein phosphatase 2A catalytic subunit beta; ***Ccng1;*** Cyclin G1; ***Pcaf;*** P300/CBP-associated factor
Integrin signaling	6	14	***Itga7, Itgb1, Itgal;*** Integrins (alpha-7 light, beta-1, alphal); *Fyn;* Proto-oncogene tyrosine-protein kinase Fyn; ***Src;*** Proto-oncogene tyrosine-protein kinase Src; ***RhoE;*** Rho-related GTP-binding protein; ***Mapk4, 13;*** Mitogen-activated protein kinases 4, 13
Hedgehog signaling	2	4	***Csnk1a1, Csnk1g3;*** Casein kinase 1; ***Prkar1;*** cAMP-dependent protein kinase type I-alpha regulatory subunit; ***Ubr5;*** E3 ubiquitin protein-ligase UBR5
Ubiquitine proteasome	4	8	***Ube2a;*** Ubiquitin carrier protein; ***Ube2n;*** Ubiquitin-conjugating enzyme E2 N; ***Uba1;*** Ubiquitin-activating enzyme E1; *Psmd2,6,12;* 26S proteasome non-ATPase regulatory subunit 2,6,12
Carnitine metabolism	1	1	***Amacr;*** Alpha-methylacyl-CoA racemase
Parkinson disease	10	9	***Csnk1a1;*** Casein kinase 1; ***Hspa8, Hspa1b, Hspa1l;*** Heat shock 70 kDa proteins (8, 1b, 1l); ***Psma1, Psma4, Psmb3;*** Proteasomes (alpfa type-1, alpha type-4, 1L)
Alzheimer disease – amyloid secretase	3	9	***Mapk4, Mapk13;*** Mitogen-activated protein kinases 4, 13; ***Pcsk1;*** Neuroendocine covertase 1; ***Pkn1, Pkn2;*** Serine/threonine kinase N1, N2
Inflammation mediated by chemokine and cytokine signaling	8	12	***Stat3;*** Signal transducer and activator of transcription 3; ***Nfkbia;*** NF-kabba-B inhibitor alpha; ***Grk6;*** G protein-coupled receptor kinase 6; ***Adcy2;*** Adenylate cyclase type 2

CMS-V, CMS vehicle; Esc-R, CMS escitalopram responders; Esc-NR, CMS escitalopram non-responders.

## Results

### Sucrose consumption

The sucrose consumption test was applied to assess stress-induced anhedonic-like behavior and antidepressant-induced recovery. Four weeks exposure to CMS resulted in significantly decreased sucrose consumption (*p*<0.001) when comparing all stress exposed rats (*n* = 249, anhedonic-like, intermediate and stress-resilient rat groups) to unchallenged controls (*n* = 80), (Figure 1A). Similarly, four weeks of antidepressant treatment with escitalopram resulted in significantly increased sucrose consumption (*p*<0.05) when comparing all escitalopram-treated anhedonic-like rats (both responders and non-responders, n = 18) with vehicle-treated CMS-rats (n = 9) (Figure 1B).

Figure 1C shows the sucrose intake (indexed to stress baseline) for the three experimental groups; CMS vehicle (CMS-V), CMS escitalopram responders (Esc-R), and CMS escitalopram non-responders (Esc-NR). The groups were defined according to changes in sucrose intake in response to exposure to chronic mild stressors and chronic antidepressant treatment with escitalopram. Antidepressant treatment proceeded for four weeks concurrently with stress exposure. As treatment progressed, chronic treatment with escitalopram reversed the decrease in sucrose intake in about 50% of the treated rats (responders).

MANOVA analysis on escitalopram treatment effects revealed significant differences over time (*F*_8,42_ = 3.96, *P* = 0.001), when comparing CMS-V (n = 9) to treatment responders (n = 9) and treatment non-responders (n = 9). Significant effects of drug treatment was present after one week of escitalopram administration (not shown) and retained in the entire period of treatment (*F*_2,24_ = 14.11, *P*_ANOVA_ <0.001, *P*_post hoc_ <0.001) Significant segregation into drug responders and drug non-responders was achieved after three weeks of escitalopram treatment (not shown) (*F*_2,24_ = 14.11, *P*_ANOVA_ <0.001, *P*_post hoc_  = 0.001).

### Transcriptomic analysis

The laser-capture microdissected LHb RNA was subjected to microarray analysis to identify transcriptomic changes related to depression and antidepressant treatment. 19.010 genes were up regulated after CMS while 12.129 genes were down regulated after CMS (Table 7). Similarly, 15.334 genes were up regulated after chronic treatment with escitalopram, while 15.805 genes were down regulated. However, this was also the case for rats responding to escitalopram treatment (Esc-R). On the contrary, escitalopram non-responders (Esc-NR) demonstrated treatment-associated up regulations of 10.441 genes while 20.698 genes were down regulated after antidepressant treatment. Ratios of up- and down regulations were similar also when focusing at the most regulated genes as illustrated in table 7.

**Table 7:** Gene regulations after CMS and antidepressant treatment.

Comparison strategy	Up regulation		Down regulation	
	All genes	Most regulated genes*	All genes	Most regulated genes*
**After stress (CMS-V vs. U-V)**	19010	8115	12129	5861
**After treatment (Esc-R vs. CMS-V)**	15334	6411	15805	7565
**After treatment (Esc-NR vs. CMS-V)**	10441	4562	20698	9414

All genes denote all probes (approx. 31.000) on the cDNA microarray chip. * Most regulated genes are calculated as standard deviation/mean (individual gene transcript intensity) > standard deviation/mean (all gene transcript intensities). U-V, unchallenged control rats; CMS-V, CMS vehicle; Esc-R, CMS escitalopram responders; Esc-NR, CMS escitalopram non-responders.

### Hierarchical clustering analysis

**Figure 2:**
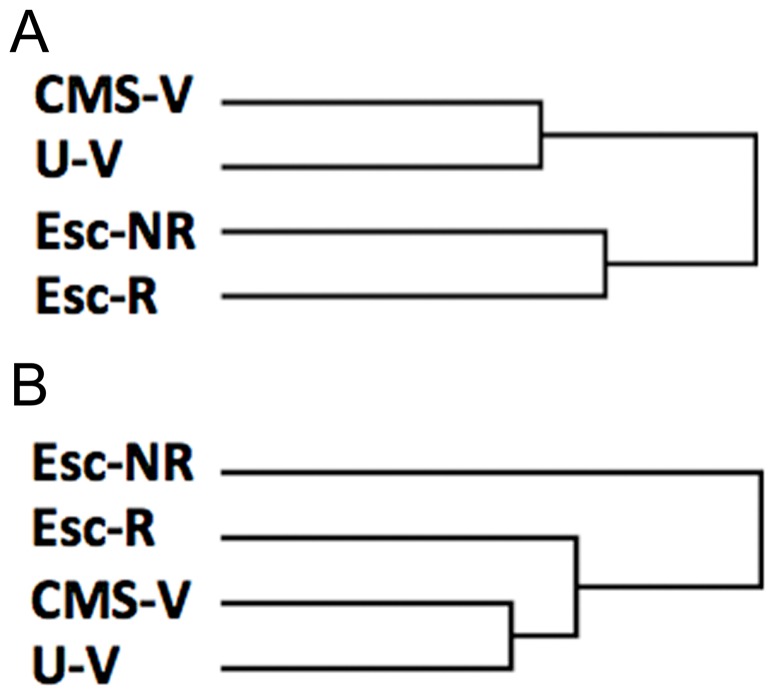
Hierarchical clustering analysis. (A) Based on all probes represented on the cDNA microarray chip showing a clear differentiation between groups receiving antidepressant treatment (Esc-R + Esc-NR) or no treatment (U-V, CMS-V). (B) Based on most regulated genes (approx. 14.000 genes – defined as gene transcripts having a standard deviation/mean (individual gene transcript intensity) > standard deviation/mean (all gene transcript intensities)), showing a clear divergence of escitalopram non-responders (Esc-NR). U-V, unchallenged control rats; CMS-V, CMS vehicle; Esc-R, CMS escitalopram responders; Esc-NR, CMS escitalopram non-responders.

The TreeView cluster analysis in Figure 2A was based on all probes on the microarray chip representing about 31.000 genes while the cluster analysis in figure 2B was based on the most regulated genes of the microarray data (i.e. standard deviation for a specific gene transcript normalized with mean expression level for the same gene is expected to be higher than the standard deviation normalized with the mean expression level for all genes). The global clustering analysis based on all genes indicated a clear differentiation between groups receiving antidepressant treatment or no treatment. However, clustering according to hedonic status was not evident from this cluster analysis. When including only the most regulated genes among the four groups in the analysis, treatment-associated effects appears, thus segregating rats responding and not responding to antidepressant treatment. Escitalopram non-responders are thus markedly diverging from all other groups in line with the results from the transcriptomic analyses (Table 7).

### Venn diagrams

**Figure 3:**
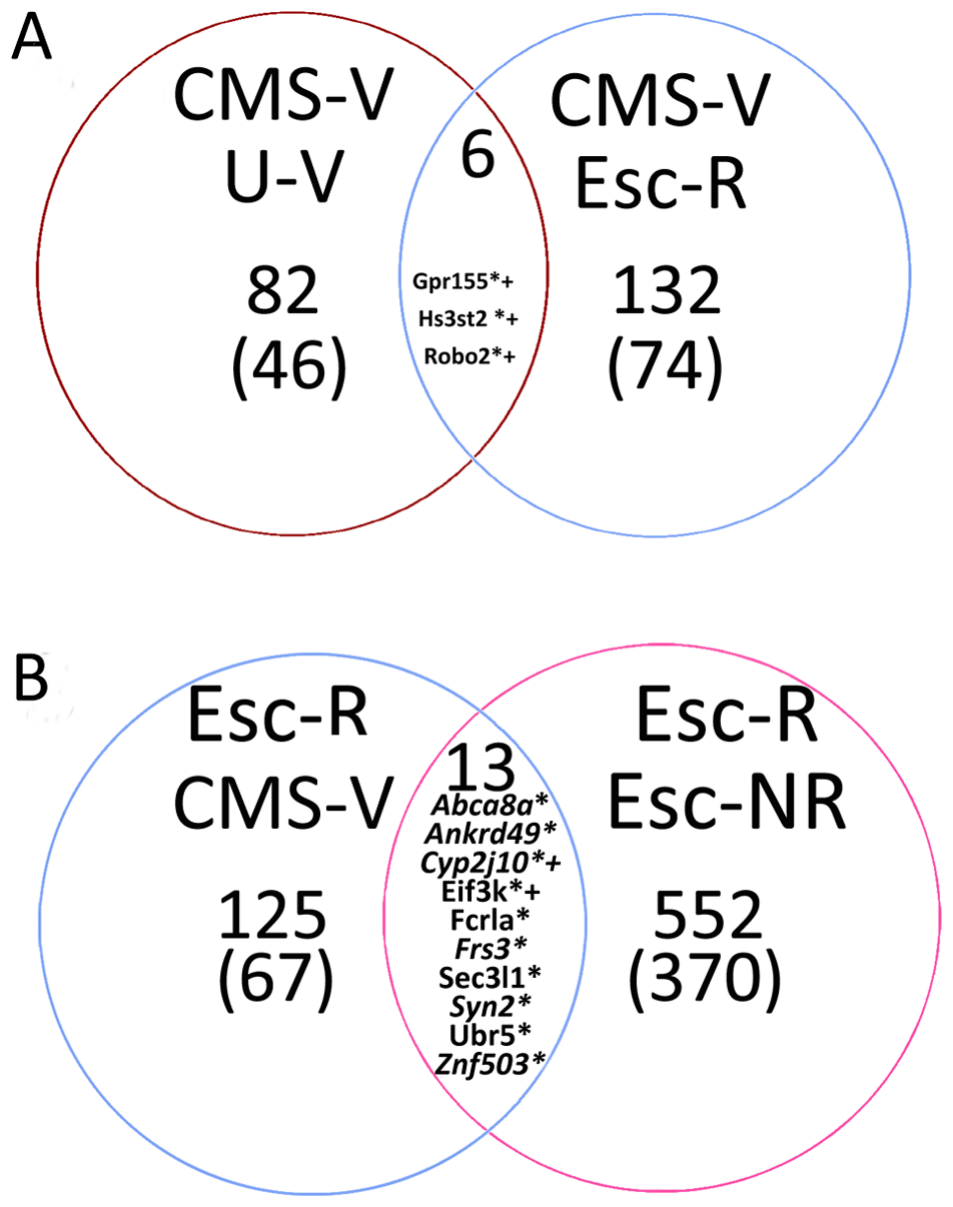
Venn diagrams for significant gene regulations associated to anhedonia and treatment recovery . (A) Associated gene differences in comparisons between vehicle-treated anhedonic-like (CMS-V) versus unchallenged control groups (left panel) and CMS-V versus treatment responders (right panel); (B) associated gene differences according to response to treatment between escitalopram responders versus CMS-V (left panel) and versus treatment non-responders (right panel). Results in parentheses indicate amount of genes identified in the NCBI database. All identified genes present in the intersection areas are listed by gene symbols. U-V, vehicle treated unchallenged control rats; Esc-R, CMS escitalopram responders; Esc-NR, CMS escitalopram non-responders. * – significant changes by qPCR for left side comparisons; + – significant changes by qPCR for right side comparisons.

First, we indentified genes involved in both the development of the anhedonia-like condition and antidepressant treatment-associated recovery. Four independent comparisons were performed to approach gene differentiation. Venn diagrams illustrated in Figure 3 demonstrate the models for comparison of genes involved in aforementioned processes. There are 88 genes differentiating CMS and stress unchallenged groups; 49 of them are identified in the NCBI database (Figure 3A and S2A, left panels). Comparison between CMS and treatment responder groups revealed changes in 138 genes, 77 of which are identified in the NCBI database (Figure 3A and S2A, right panels). Intersection area of Venn diagram in Figure 3A contains 6 genes and 3 of them are identified in NCBI database and up-regulated in both comparisons. Antidepressant treatment responders and non-responders differ in 565 genes, where 380 were identified in the NCBI database. A list of 46 genes from this comparison is shown in Figure S2B. Two comparisons describing differences in gene expression specific to response to antidepressant treatment revealed 13 common genes (Figure 3B). Ten of them are identified. *Fcrla, Eif3k, Sec3l1*, and *Ubr5* were up-regulated in comparison to both CMS and escitalopram non-responders groups; levels of *Abca8a, Ankrd49, Cyp2j10, Frs3, Syn2, Znf503* were lower in escitalopram responders compared to CMS and non-responders groups.

### Real-time qPCR

**Figure 4:**
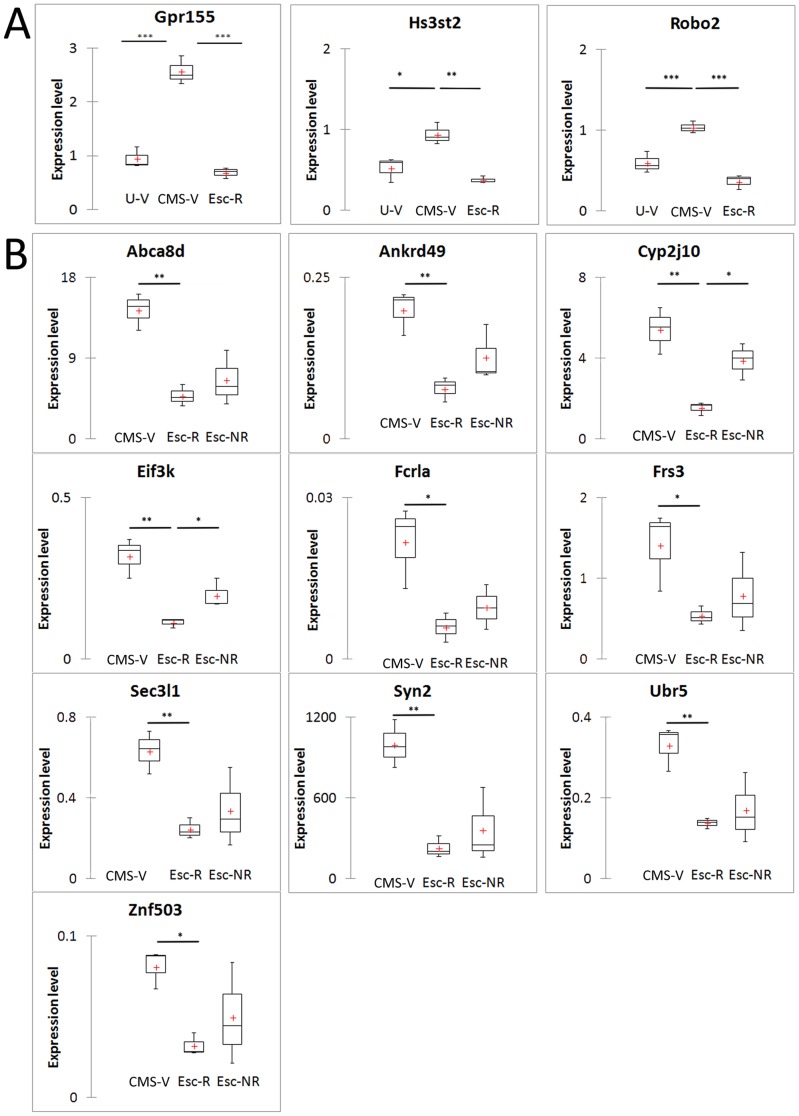
Expression levels of the NCBI identified genes confirmed by qPCR. (A) Genes changed in comparisons between anhedonic-like versus unchallenged controls and anhedonic-like versus treatment responders groups. (B) Genes regulated in comparisons between treatment responders versus anhedonic-like and treatment responders versus treatment non-responders groups. U-V, vehicle treated unchallenged control rats; CMS-V, vehicle treated anhedonic-like rats; Esc-R, CMS escitalopram responders; Esc-NR, CMS escitalopram non-responders. Red crosses indicate arithmetical mean values; *, **, *** – p<0.05, 0.01, 0.001, respectively, by Dunnett's ANOVA post-test: A – in comparison with CMS-V; B – in comparison with Esc-R.

Secondly, 13 genes from intersection areas of both Venn diagrams (Figure 3) were used in qPCR experiments to confirm changes in gene expression discovered by microarray analysis. Results presented in Figure 4A demonstrate up-regulation of three genes in CMS rats compared to both unchallenged controls and escitalopram responders and thus confirming findings discovered in the microarray analysis. Figure 4B demonstrates qPCR revealed changes in expression in treatment responders versus anhedonic-like rats (CMS-V) and treatment non-responders rats, respectively. Interestingly, qPCR quantification resulted in relatively large deviations in all genes in treatment non-responders group; therefore the 95% of significance in comparison between groups was achieved only for *Cyp2j10* and *Eif3k* genes, but the directions in most gene regulations were the same as in results of microarray analysis. Groupwise relationships according to the response to stress and response to antidepressant treatment are shown in Figure S3. CMS led to up-regulation of all tested genes according to the stress-unchallenged controls and positive response to antidepressant treatment was associated with gene expression recovery (Figure S3A). Comparison according to the response to treatment revealed a strong up-regulation of tested genes in both CMS and treatment non-responders groups with confirmed significant changes for most of the genes in the CMS group (Figure S3B).

### Biological pathway analysis

Due to relatively small stress- and treatment-associated gene regulations at single gene levels, significantly regulated genes (*p*<5%) were analyzed according to implications in biological pathways. As illustrated in table 3 and table 5, most pathways associated with recovery and treatment response belong to the two categories of pathways; growth factor receptor signaling and G protein–coupled receptor signaling. Furthermore, stress and escitalopram treatment affect additional intracellular pathways like integrin signaling, hedgehog signaling, and chemokine and cytokine signaling. The genes listed in table 4 and table 6 constitutes only a fraction of the genes represented in the pathways. Genes listed in the tables are randomly chosen from the entire set of genes in the individual pathways.

## Discussion

In the present study we used the validated rat CMS model of depression to mirror symptoms of human depression and to investigate molecular correlates of depression and antidepressant treatment in the lateral habenula. In this study there was a general effect of four weeks of mild stress exposure on sucrose intake with a mean decrease of 29% (Figure 1A). As previously reported, rats segregate into two groups when exposed to CMS, one group sensitive to CMS, thus reflecting anhedonic-like behavior, and one group resilient to CMS [18], [25]–[27]. Another unique feature of the CMS model of depression is that only a fraction of the drug-treated rats respond to antidepressants, thus leaving the remaining rats in the anhedonic-like state (CMS-Esc-NR). This phenomenon is well known from the clinic as only a fraction of the depressive patients respond to antidepressant medication. The proportion of drug responding/non-responding rats constitutes approximately 50%, thus allowing us to investigate molecular mechanisms of drug-induced recovery and therapy refraction, respectively. This unique individual treatment response as seen in the present study is reproducible in all our CMS studies [19], [23]–[25], [27], and has not been reported in CMS studies elsewhere [17], [20], [23], [33]. The proportion of escitalopram responders in the CMS model of depression correlates to clinical response rate ranging from approximately 60% to 85% in patients suffering from moderate or severe depression disorders [34], [35]. The placebo effect observed in clinical studies is 25–35% and may explain the higher response rates observed in clinical studies when compared to animal studies [36]. As deduced from the present CMS experiment, there is a general effect of antidepressant treatment with escitalopram after four weeks of treatment with a mean increase in sucrose intake of 43% (Figure 1B). This increase is even more pronounced in the escitalopram responder group (with a mean increase in sucrose intake of 73%) as illustrated in Figure 1C. On the other hand, antidepressant treatment had no significant effect on rats not responding to treatment illustrated by unchanged drinking behavior in the sucrose consumption test. Escitalopram displayed ameliorating effects on CMS-induced anhedonia (in the responder group – Esc-R) after one week of treatment (not shown), while full recovery was obtained after four weeks of treatment. The variable treatment response seen is further validated in the hierarchical clustering analysis based on the most regulated genes (approximately 14.000 genes), where the escitalopram non-responder group, in specific, diverges from all other CMS-generated phenotypes, indicating major molecular aberrations in treatment-resistant rats. Thus, in addition to modeling recovery, the CMS model of depression also appears to imitate human therapy refraction.

In the present study, a genome wide microarray analysis in CMS- and/or treatment-induced phenotypes was performed to assess effects on global transcriptomic profiles. This approach was chosen to quantitatively assess stress-related behaviors and antidepressant treatment of genes in order to investigate the molecular role of the LHb. The aim of the study was to narrow down the set of genes that were differentially regulated by stress and antidepressant treatment. Although many genes were found to be regulated most of them were only modestly regulated (*p*<0.05 and maximal fold changes of 30–40%). Therefore, differentially regulated genes were investigated collectively according to biological pathways. Stress- and treatment-associated gene regulations at this level is a common phenomenon confirming the statement from Mirnics et al. [37], that gene expression changes in psychiatric traits are small and that psychiatric diseases may result from cumulative subtle differences. Additionally, we have previously identified small gene regulations in a similar transcriptomic study investigating CMS- and treatment-induced changes in a hippocampal subregion [25]. Considering the small gene regulations in the present study and the high risk for false positives of individual genes, our strategy was to search for biological pathways implicated in depression and antidepressant treatment. We believe that clusters of genes pointing towards specific biological processes is the most convincing way to identify targets related to recovery and drug response under the present circumstances.

**Figure 5:**
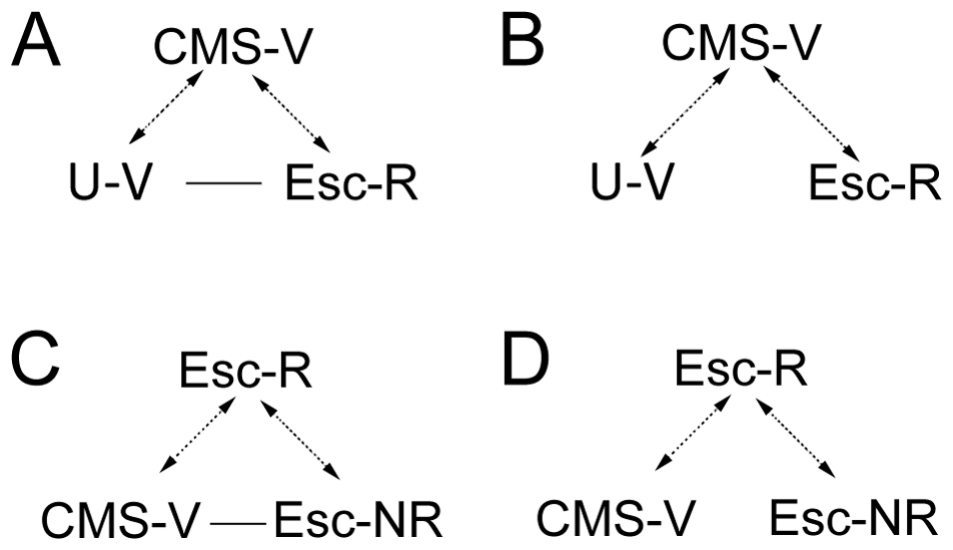
Relationships between groups built up for PANTHER analysis. Statistical enrichment of biological pathways was assessed by binomial statistics within the PANTHER system. (A and B) Relationships for classification of pathways implicated in depression and recovery; (C and D) Relationships for classification of pathways implicated in treatment response. Dashed lines indicate significant differences in pathways between groups (*p*<0.05), regular lines indicate no differences in pathways between groups. U-V, unchallenged control rats; CMS-V, CMS vehicle; Esc-R, CMS escitalopram responders; Esc-NR, CMS escitalopram non-responders.

Constraints were applied for analysis of differential pathway regulation profiles and thus for the identification of genes/pathways involved in depression etiology (anhedonia) and recovery (Figure 5). Hence, pathways were prioritized if they were significantly regulated after exposure to CMS, and concomitantly also significantly affected by chronic treatment with escitalopram in the responder group (Esc-R) (Table 3). Using the applied constraint reduces the risk of false positives and allows us to focus on treatment effects specifically associated with recovery, thus excluding the non-specific (non-anhedonia-related) drug effects of escitalopram. According to the applied constraints we found 11 distinct pathways to be involved in the etiology/recovery of depression, two of which are affected when comparing unchallenged controls (U-V) with healthy drug-recovered (Esc-R) rats (Table 3). Among pathways involved in depression pathology/recovery, we found several intracellular signal transduction pathways. One such pathway category is growth factor receptor signaling (vascular endothelial growth factor (VEGF) signaling, epidermal growth factor (EGF) signaling and angiogenesis). Among growth factors, more specifically, brain-derived neurotrophic factor (BDNF) has been suggested as a strong candidate modulating stress-associated pathology and antidepressant treatment. This have led to the development of a neurotropic hypothesis of depression suggesting that stress-induced decreases of BDNF expression, and possibly other growth factors, contributes to the progression of depression and that up-regulation of BDNF plays a role in the actions of antidepressant treatment [38]–[40]. VEGF is another growth factor, which has gained lots of attention during the last decade with respect to depression and antidepressant treatment. VEGF was first identified as an angiogenic factor essential for the formation of new blood vessels in physiological and pathological processes [41], [42]. In addition to stimulating endothelial cell proliferation, migration, and survival recent studies established a neurotrophic and neuroprotective role of VEGF [43], [44]. Just like BDNF, VEGF also stimulates adult neurogenesis and a decreased level of neurogenesis as a consequence of chronic stress associates with decreased VEGF levels [43], [45]. Furthermore, exposing rats to the CMS model of depression resulted in decreased VEGF expression in the hippocampus [23]. On the other hand multiple classes of antidepressants and ECS increase the expression of VEGF [46]-[48] and it has been show that blockage of VEGF expression blocks the beneficial effects of antidepressants in animals subjected to unpredictable chronic mild stress [49], [50]. In the present study we found that the VEGF signaling pathway was affected during depression and antidepressant treatment. Genes affecting this pathway includes *vegfa* and *Flt1 (VEGF receptor 1),* and downstream intracellular pathways of the growth factor receptor signaling pathway, like protein kinase C (*Prkca, Prkd3 and Prkci)* and signal transducers and activators of transcription 3, 4, 6 (*Stat3,Stat4, Stat6)*(Table 4). Another prominent intracellular pathway identified in the present study with respect to depression and antidepressant treatment is the Wnt signaling pathway. Wnt's are secreted glycoproteins that signal through the frizzled (Fz) receptors which couple to several signaling cascades. Wnt signaling is crucial for embryonic development and disruption of this cascade has been associated with various neurodevelopmental and neuropsychiatric disorders [51], [52]. More specific, a role for the Wnt signaling pathway in depression-related behavior has been reported in several studies. Stress is associated with imbalances of Wnt signaling molecules and several of these molecules are regulated by various classes of antidepressants [53]. Genes affecting the Wnt pathway in the present study includes the intracellular signaling molecules *Axin1*, secreted frizzeled-related protein 2 (*sfrp2*) and segment polarity protein dishelveled homolog DVL-1 (*dvl1*) (Table 4). In fact, Wnt's and growth factors/neurogenic factors share common intracellular downstream cascades, targeting both the mitogen-activated protein kinase/extracellular signal-regulated kinase (MAPK/ERK) and phosphatidylinositol-3-kinase (PI3K) cascades. Both of these intracellular pathways were shown to be disrupted in subjects with depression and in animal models of depression [53]. Integrins, transmembrane receptors that mediate the attachment between a cell and the tissue that surrounds it, also appeared as a pathway of interest under the present constraints. Research combining depression and integrin signal transduction is scarce; however, intracellular signals via integrins are related to neuroplastic mechanisms like cell growth and division, cellular differentiation and apoptosis [54]. The integrin-mediated signaling pathway converges with intracellular pathways mediated by growth factors, resulting in transcriptional stimulation via the MAPK/ERK cascade. In the present study, changes in integrin-mediated signaling was mediated via an integrin ligand (fibronectin, *Fn1*), integrin receptors (integrin alpha-7 light, *Itga7* and integrin alpha-M, *Itgam*), and downstream signaling molecules like proto-oncogene tyrosine-protein kinase Fyn (*Fyn*) and son of sevenless homolog 1 (*Sos1*) (Table 4). All three pathways mentioned in the present study: growth factor receptor signaling, Wnt signaling and integrin-mediated signaling, converge on activating the transcription factor cAMP response element-binding protein (CREB), which is known to regulate the transcription of several genes implicated in depression pathology and treatment response [53], [54]. Furthermore, CREB has repeatedly been shown to be modulated in depressive-like states and after antidepressant treatment [55]. In fact, we also found that transcriptional regulation was significantly regulated as a pathway in the present study, including the transcription factor processed cyclic adenosine monophosphate (cAMP)-responsive element-binding protein 3-like protein 2 (*creb3l2*), indicating depression and treatment-associated changes even at a transcriptional level of signal transduction (Table 4).

The segregation of drug-treated CMS rats into responder (Esc-R) and non-responder (Esc-NR) groups, allows us to search for molecular pathways implicated in the individual treatment response seen in the CMS model of depression. Pathways of interest will thus reflect mechanisms affected by antidepressant treatment (CMS-V versus Esc-R), but also mechanisms showing divergence between rats responding to escitalopram treatment and rats not responding to antidepressant treatment (Esc-R versus Esc-NR; Figure 5C-D). According to the applied constraints we found 24 distinct pathways to be involved in treatment response, seven of which are affected when comparing anhedonic-like rats (CMS-V) with escitalopram non-responders (Esc-NR) rats (Table 5). A major part of pathways implicated in treatment response belongs to the G-protein-coupled receptor-signaling pathway. Molecular pathways represented in this pathway category includes metabotropic glutamate receptor signaling, dopamine receptor-mediated signaling, 5HT1 type receptor-mediated signaling and GABA-B receptor signaling, among others (Table 5) – all mediating their physiological functions via G-proteins. Common for this category of pathways is that ligand binding to the G protein coupled receptors activate or inhibit the adenylyl cyclase (AC)-cAMP signal transduction pathway. AC is the critical enzyme to catalyze cAMP syntese. cAMP is the ubiquitous intracellular second messenger, which stimulates activities of cAMP-dependent protein kinases (e.g. protein kinase A – PKA) and CREB. In line with CREB alterations during depression, PKA levels are found to be reduced in depressed suicide subjects and in animal models of depression [56]. Evidence from a number of different studies has demonstrated that antidepressant treatments up-regulate the AC-cAMP signal transduction pathway, including increased levels of Gαs coupling to AC and increased levels of PKA leading to increased CREB function [57]. In fact, in the present study we found that *Creb1* is affected by antidepressant treatment as well as cAMP-dependent protein-kinase type-I-alpha regulatory subunit (*Prkar1a*), adenylate cyclase 2, 3 and 5 (*Adcy2*, *Adcy3*, *Adcy5*) and several G proteins; *Gnai3*, *Gnb1*, *Gng10* (Table 6). Additionally, we found other intracellular pathways implicated in treatment response, like hedgehog signaling, integrin signaling, growth factor receptor signaling, and chemokine and cytokine signaling (Table 5).

In general, the present microarray study led to molecular findings associated with depression and antidepressant treatment in the LHb. Due to low regulations at gene level, we applied a strategy searching for pathways implicated in the development and recovery from depression. Pathways involved in intracellular signal transduction are highly represented in the present study. As deduced from above, alterations in intracellular signaling and components involved in these processes have repeatedly been associated with the disorder of depression and its treatment. It is known that intracellular signaling is a mediator of neuroplasticity and molecular mechanisms of neuroplasticity indeed overlap molecular mechanisms of depression and antidepressant response. However, most depression-related studies focus on research on the frontal cortex or the hippocampus as dysfunctions in both of these brain regions are highly associated with depression and treatment. To our knowledge, the present study is the first to investigate molecular mechanisms of depression-like behavior and treatment in the LHb of rats exposed to an animal model of depression. Deduced from our study, it seems that stress and antidepressant treatment primarily modulate signal transduction pathways in LHb, leading us to speculate that increased intracellular signaling contributes to increases in neuronal activities during depressive-like states. The conclusions assumed from the present study are speculative and based on pathway analyses on significant gene alterations. In order to confirm the present results, it is necessary to validate that the individual intracellular signaling systems in fact are disturbed under depression and antidepressant treatment. Monoaminergic systems like serotonin, dopamine, and noradrenalin neurotransmitter systems are deregulated in depressive-like states. The LHb directly innervates these transmitter systems, indicating a central role for this brain region during depression and antidepressant treatment. A dysfunction of afferent and efferent LHb innervation might thus create imbalances in monoaminergic neurotransmission leading to depressive-like states or vice versa. Unraveling the role of the LHb in depression and treatment might lead us to new molecular targets involved in depression pathology and novel treatment strategies.

## Supporting Information

Figure S1 Coronal slice image with the selected lateral habenula area. DG, dentate gyrus; D3V, Dorsal third ventricle; mHb, medial habenula; LHb, lateral habenula.
(TIF)

Figure S2 Venn diagrams of NCBI identified genes. (A) In between groups comparisons for CMS-V versus U-V and CMS-V versus Esc-R; (B) In between groups comparisons for Esc-R versus CMS-V and Esc-R versus Esc-NR. In **bold** font are written symbols for genes up-regulated in the current comparison, in *italic* font are written symbols of genes down-regulated in the current comparison. * – significance of changes confirmed by qPCR for left-side comparison; + – significance of changes confirmed by qPCR for right-side comparison. CMS-V, vehicle treated anhedonic-like group; U-V, vehicle treated CMS unchallenged group; Esc-R, escitalopram treatment responders group; Esc-NR, escitalopram treatment non-responders group.
(TIF)

Figure S3 Stress and anti-depressant effects of genes measured by qPCR. (A) Stress-induced up-regulation of tested genes demonstrated as a ratio of gene expression between CMS-V and U-V groups; treatment-induced recovery demonstrated as a ratio of gene expression between Esc-R and U-V groups. (B) According to response to escitalopram treatment (ratio to Esc-R) all tested genes were up-regulated in both CMS-V and Esc-NR groups. *, **, *** – p<0.05, 0.01, 0.001, respectively, by Dunnet's ANOVA post test: A – in comparison with U-V; B – in comparison with Esc-R. CMS-V, vehicle treated anhedonic-like group; U-V, vehicle treated CMS unchallenged group; Esc-R, treatment responders group; Esc-NR, treatment non-responders group.
(TIF)

Table S1 Database related characteristics for 13 genes used to confirm gene expression results by qPCR.
(DOCX)
