# Supplementary figures and images for: Molecular Profiling of the Lateral Habenula in a Rat Model of Depression
Source: PLoS One. 2013 Dec 5;8(12):e80666. doi: 10.1371/journal.pone.0080666 (PMC3855087; doi:10.1371/journal.pone.0080666)

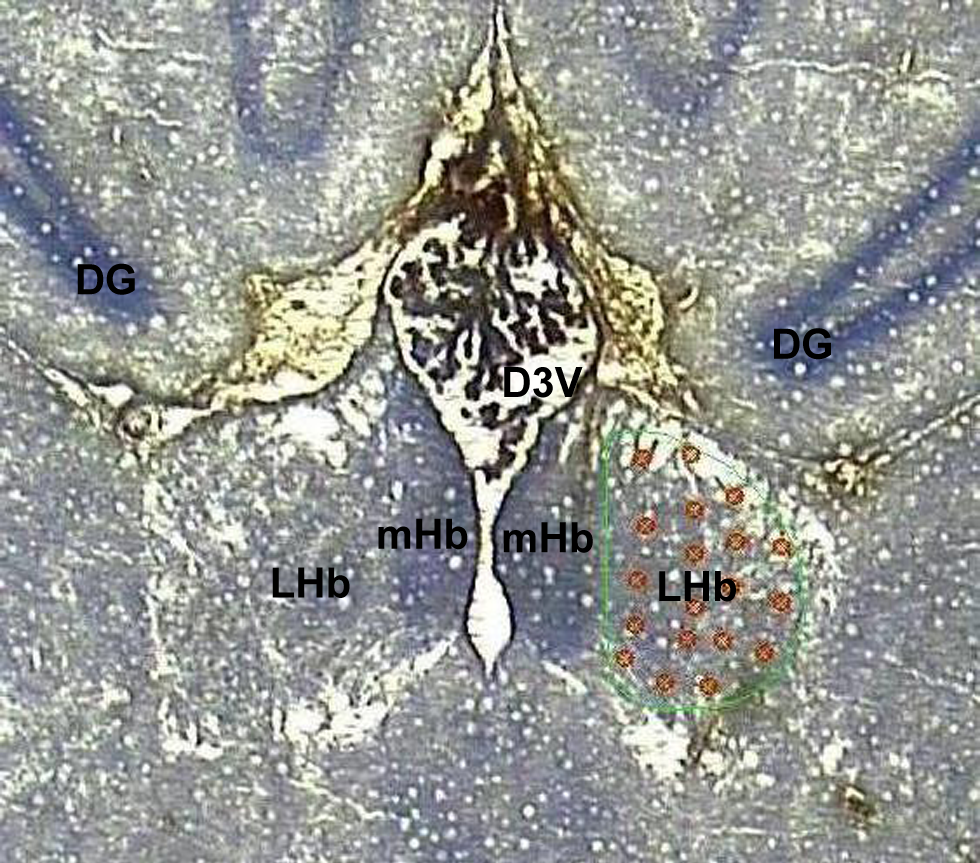

Supplement: Figure S1 — Coronal slice image with the selected lateral habenula area. DG, dentate gyrus; D3V, Dorsal third ventricle; mHb, medial habenula; LHb, lateral habenula. (TIF) [file pone.0080666.s001.tif]

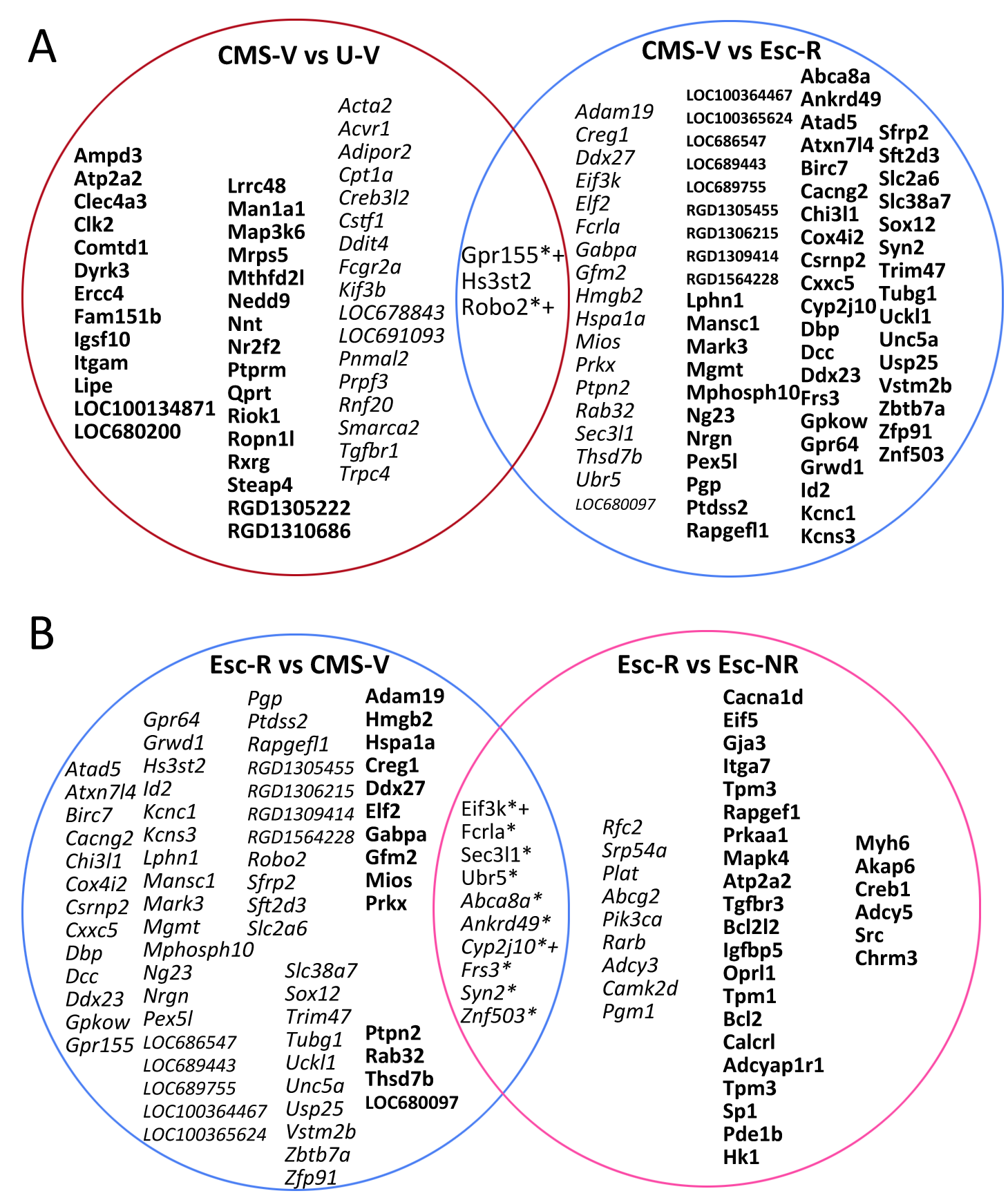

Supplement: Figure S2 — Venn diagrams of NCBI identified genes. (A) In between groups comparisons for CMS-V versus U-V and CMS-V versus Esc-R; (B) In between groups comparisons for Esc-R versus CMS-V and Esc-R versus Esc-NR. In bold font are written symbols for genes up-regulated in the current comparison, in italic font are written symbols of genes down-regulated in the current comparison. * – significance of changes confirmed by qPCR for left-side comparison; + – significance of changes confirmed by qPCR for right-side comparison. CMS-V, vehicle treated anhedonic-like group; U-V, vehicle treated CMS unchallenged group; Esc-R, escitalopram treatment responders group; Esc-NR, escitalopram treatment non-responders group. (TIF) [file pone.0080666.s002.tif]

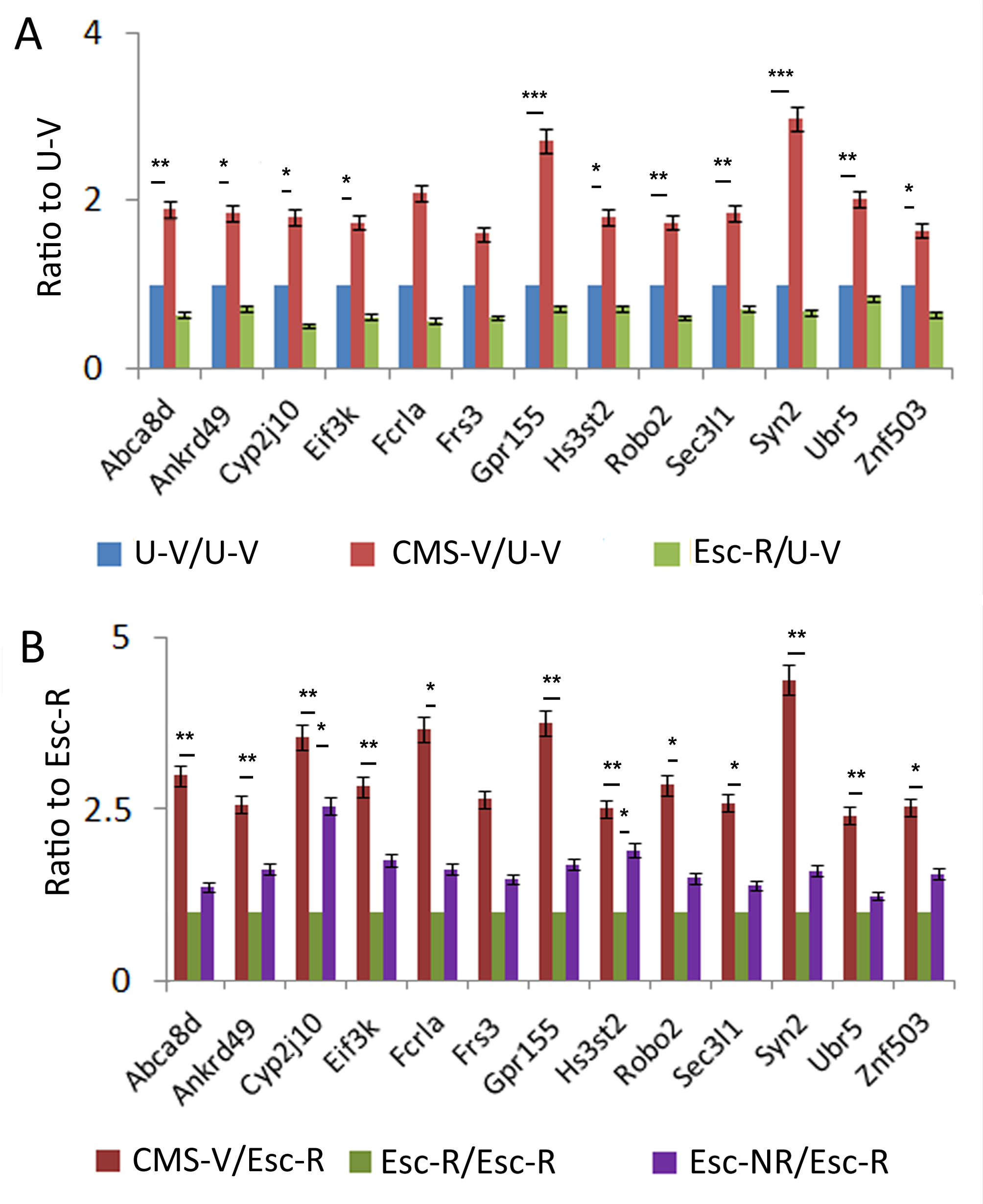

Supplement: Figure S3 — Stress and anti-depressant effects of genes measured by qPCR. (A) Stress-induced up-regulation of tested genes demonstrated as a ratio of gene expression between CMS-V and U-V groups; treatment-induced recovery demonstrated as a ratio of gene expression between Esc-R and U-V groups. (B) According to response to escitalopram treatment (ratio to Esc-R) all tested genes were up-regulated in both CMS-V and Esc-NR groups. *, **, *** – p<0.05, 0.01, 0.001, respectively, by Dunnet's ANOVA post test: A – in comparison with U-V; B – in comparison with Esc-R. CMS-V, vehicle treated anhedonic-like group; U-V, vehicle treated CMS unchallenged group; Esc-R, treatment responders group; Esc-NR, treatment non-responders group. (TIF) [file pone.0080666.s003.tif]
