# Supplementary material for: Molecular Profiling of the Lateral Habenula in a Rat Model of Depression
Source: PLoS One. 2013 Dec 5;8(12):e80666. doi: 10.1371/journal.pone.0080666 (PMC3855087; doi:10.1371/journal.pone.0080666)
Supplement: Table S1 — Database related characteristics for 13 genes used to confirm gene expression results by qPCR. (DOCX) [file pone.0080666.s004.docx]

## Table S1. Database related characteristics for 13 genes used to confirm gene expression results by qPCR.

| **Gene Symbol** | **Transcript Accession** | **Context Sequence** | **Gene Name** |
| --- | --- | --- | --- |
| Syn2 | NM_001034020.1; NM_019159.1; M27925.1; M27926.1 | CATGGGTGTTTGCTCAGATGGTGGC | synapsin II |
| Hs3st2 | NM_181370.2; AY240873.1; BC128721.1 | GGCTGGACTGGTACAGGAGCCTGAT | heparan sulfate (glucosamine) 3-O-sulfotransferase 2 |
| Sec3l1 | NM_001024770.1; BC097392.1 | TCTGCGTCAATGGCTGAAGCAGAAG | SEC3-like 1 (S. cerevisiae) |
| Cyp2j10 | NM_001134980.1; BC168751.1 | TCAGAAGTTTGTGAAGAAGTATGGA | cytochrome P450, family 2, subfamily j, polypeptide 10 |
| Abca8a | XM_221100.6 | GCCAAGTGCTGGACAGGTGCAGCTG | ATP-binding cassette, subfamily A (ABC1), member 8a |
| Eif3k | NM_001106242.1 | TGCTCGGAGACCTGACTGACAACCA | eukaryotic translation initiation factor 3, subunit K |
| Fcrla | NM_001100682.1; BC105902.1; BC158820.1 | AGATCCGGGTACAGGCTCTTCAGAA | Fc receptor-like A |
| Robo2 | NM_032106.1; AF182037.1 | ATTAAGAATTTACGGATTTCTGATA | roundabout homolog 2 (Drosophila) |
| Ubr5 | X64411.1 | TTTCAATGATGAGTCAGGAGAGAAT | ubiquitin protein ligase E3 component n-recognin 5 |
| Frs3 | NM_001017382.1; AY972083.1 | CAGCCCACGGGCTACACAGTCTCCA | fibroblast growth factor receptor substrate 3 |
| Gpr155 | NM_001107811.1 | TCATCGGCCTGTTTGCTAATCTCTC | G protein-coupled receptor 155 |
| Ankrd49 | NM_001126283.1; BC161982.1 | CTGCTGAAAAGAATCGGCTTGCTAC | ankyrin repeat domain 49 |
| Zfp503 | NM_001107250.1; BC166993.1 | CCCATCGAGCTGGACGCCAAGAAGA | zinc finger protein 503 |
